# Construction and Validation of a 6-Ferroptosis Related Gene Signature for Prognosis and Immune Landscape Prediction in Melanoma

**DOI:** 10.3389/fgene.2022.887542

**Published:** 2022-05-25

**Authors:** Zhanghui Yue, Jianfang Sun, Liqing Shi

**Affiliations:** Institute of Dermatology, Chinese Academy of Medical Sciences and Peking Union Medical College, Nanjing, China

**Keywords:** melanoma, ferroptosis, prognostic model, tumor-infiltrating immune cells, immunotherapy, immune checkpoints

## Abstract

Ferroptosis is a newly discovered form of non-apoptotic cell death that relies on iron-mediated oxidative damage, playing a crucial role in the progression and therapy resistance of melanoma. Hence, the potential value of ferroptosis-related genes (FRGs) as a prognostic model and therapeutic target in melanoma requires further investigation. In this study, the relationship between FRGs and melanoma was revealed by analyzing the mRNA expression profiles from The Cancer Genome Atlas (TCGA) and Gene Expression Synthesis (GEO). A 6-FRGs signature was constructed by Univariate, multivariate, and lasso Cox regression analyses in the TCGA cohort. The GEO database was used to validate the efficacy of the signature. The protein and mRNA expression level of the signature genes were examined in real-world melanoma tissues via immunohistochemical and quantificational real-time polymerase chain reaction (qRT-PCR). Functional enrichment analysis and immune-related analysis were conducted to identify the potential biological functions and pathways of the signature. Ten putative small molecule drugs were predicted by Connectivity Map (CMAP). As a result, a 6-FRGs signature was constructed to stratify melanoma patients into two risk groups. Compared with the low-risk group, patients in the high-risk group had a worse prognosis and a lower ImmuneScore. Immune-related pathways were enriched in the low-risk group. Immune Function and immune cell infiltration of the low-risk group were significantly higher than that of the high-risk group. The differential expression of these six FRGs in melanoma and adjacent normal tissues was confirmed. Moreover, higher expression of immune checkpoint molecules and a greater sensitivity to immunotherapy were observed in the low-risk group. Some small molecular drugs in the CMAP database hold the potential to treat melanoma. Overall, we identified a novel FRGs signature for prognostic prediction in melanoma. Based on the signature-related immune infiltration landscape found in our study, targeting the FRGs might be a therapeutic alternative for melanoma.

## Introduction

Melanoma is one of the most aggressive skin cancers that causes approximately 55,500 deaths globally each year, and the mortality rate is rising rapidly (Ferlay et al., 2015). Existing approaches, such as Dermoscopy, tumor-lymph node-metastasis (TNM) classification, Breslow thickness, help to diagnose and assess the prognosis of melanoma patients, but an early and accurate judgment for melanoma is still hard to reach. In the past decade, the emergence of targeted therapy and immunotherapy has considerably improved the overall survival (OS) of melanoma patients (Michielin et al., 2019). However, only half of the metastatic melanoma patients respond to checkpoint inhibitor therapy (Di Pietro et al., 2021), which might attribute to subtype bias, intra-tumor heterogeneity (Lu et al., 2021), low mutational load (Budczies et al., 2018), phenotypic plasticity, etc. (Valsecchi, 2015). Therefore, it is of clinical significance to explore potential biomarkers to assess prognosis and determine which patients will benefit from immunotherapy.

Ferroptosis, a novel cell death manner caused by iron-dependent lipid peroxidation, is characterized by oxidative modification of phospholipid membranes (Gagliardi et al., 2020). Compelling evidence has demonstrated that increased iron demand makes cancer cells more vulnerable to ferroptosis (Hassannia et al., 2019), it affects cancer resistance to certain chemotherapeutic drugs, and mediate the efficacy of immunotherapy (Lu et al., 2017). Several FDA-approved anti-cancer drugs (altretamine, sorafenib, and silica nanoparticles) were recently confirmed as ferroptosis inducers and created high expectations for the therapeutic potential of ferroptosis (Shen et al., 2021). Treatment modalities based on the combination of ferroptosis and tumor immunity activation offers potential for novel therapeutic strategy. Specifically, knockdown of GPX4, a central mediator of ferroptosis, induces renal cell carcinoma cell death with accompanying lipid ROS generation but can be rescued by the iron chelator DFO and the antioxidant vitamin E (Yang et al., 2014). Upregulation of SLC7A11, a member of cystine/glutamate antiporter, protected cancer cells from ferroptosis while SLC7A11 inhibition induced ferroptosis and enhanced cisplatin cytotoxicity in cisplatin-resistant head and neck cancer cells (Dixon et al., 2012; Roh et al., 2016). Moreover, immunotherapy triggers the ferroptosis of cancer cells, and blocking the ferroptosis pathway lowers the tumor sensitivity to immunotherapy (“Immunotherapy Activates Unexpected Cell Death Mechanism,” 2019). Recently, some ferroptosis-related prognostic models have been constructed in glioma (Liu et al., 2020; Zhuo et al., 2020), hepatocellular carcinoma (X. Du & Zhang, 2020; Huo, Cai, Guan, Liu, & Wu, 2021; Liu et al., 2021), renal cell carcinoma (Wu et al., 2020; Wang et al., 2021), etc., further indicating the prognostic value and the potential as oncologic therapeutic targets of FRGs in human cancers. However, studies on FRGs in the prognosis and immune landscape of melanoma remain limited.

In the present study, based on the differentially expressed FRGs in melanoma, we constructed a 6-FRGs signature in the TCGA database and validated the prognostic model in the GEO database. Immune-functional analysis was performed to explore the immune landscape in melanoma and the potential effects of ferroptosis in immunotherapy. Finally, we screened several small-molecular compounds could be a potential therapeutic strategy for melanoma.

**FIGURE 1:**
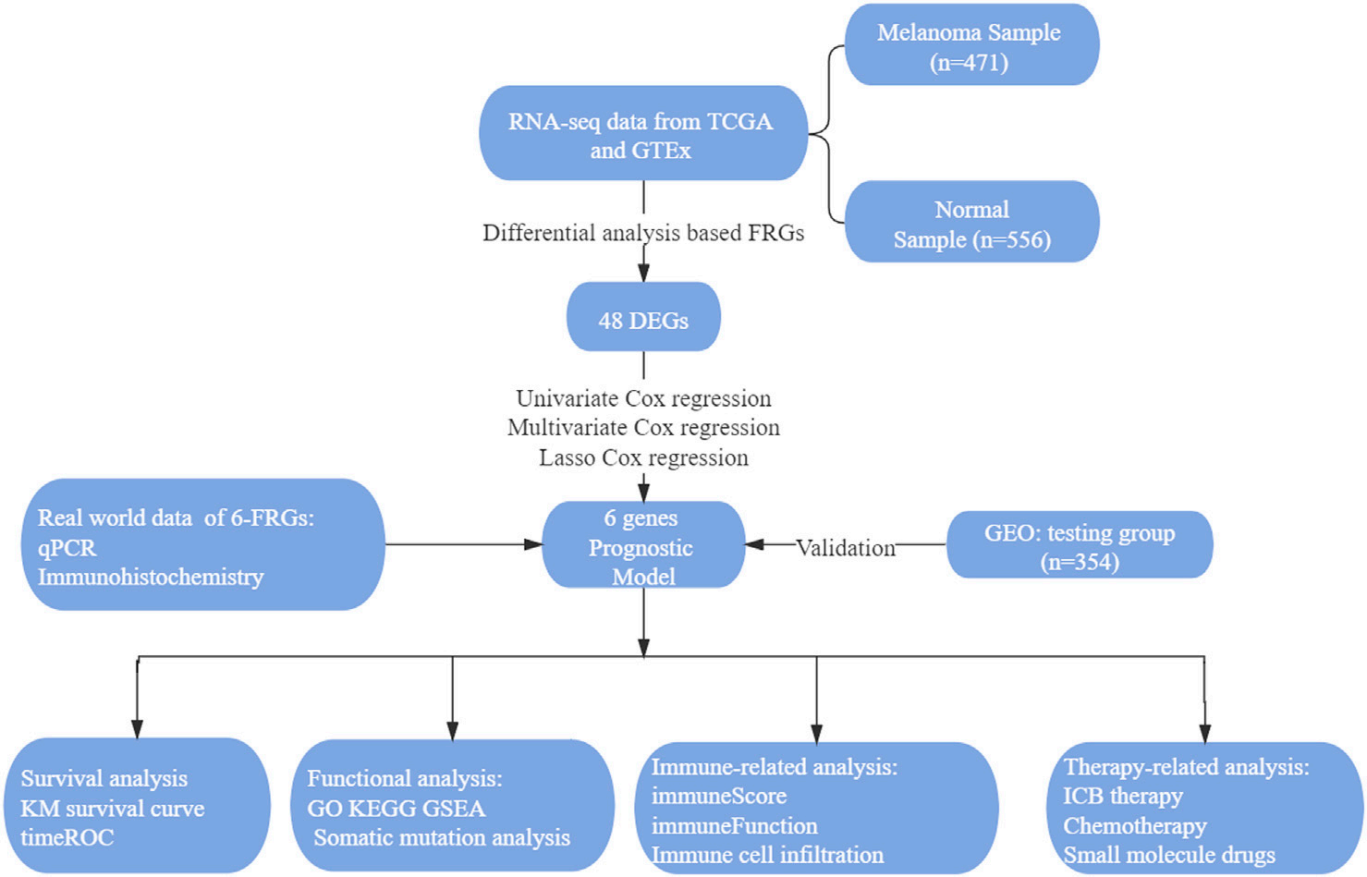
The visual flow-process diagram of this study.

The flowchart in Figure 1 shows the key steps of data procession, analysis, and validation for this study (Figure 1).

## Material and Methods

### Raw Data Acquisition

RNA-Seq data and clinical information of 470 melanoma patients were obtained from the TCGA^1^ as a training set. The normal skin samples were retrieved from the GTEx^2^. Another 332 melanoma patients’ information from GEO^3^ (data merged from GSE15605, GSE19234, GSE22154, GSE54467, and GSE65904) were used as a testing set. Batch correction was implemented when different cohorts were integrated. All the datasets above were available to the public.

### Identification of Differentially Expressed Genes

The DEGs were screened by comparing the expression of 259 FRGs (http://www.zhounan.org/ferrdb/index.html) between melanoma and normal skin tissue in the TCGA and GTEx database. We identified DEGs with | log2 fold change (log2FC) | > 1.0 and false discovery rate (FDR) < 0.05 and visualized them into volcano plots and heatmaps by the “limma” and “pheatmap” software package in R.

### Establishment of Prognostic Model

Univariate, lasso, and multivariate Cox regression analyses were performed to assess the independent prognostic value of the FRGs and construct a prognostic model. The following formula was used to calculate the RiskScore: RiskScore = ∑ni = 1 Coefi * Xi. Coefi means the coefficients, Xi means the value of six genes selected to establish the prognostic model. Then, RiskScores for all patients in the TCGA and GEO dataset were computed. The area under curve (AUC) values were calculated to evaluate the prognostic abilities of the RiskScore and other clinicopathological features. The corresponding 1 -, 3 -, and 5-year receiver operating characteristic (ROC) curves were plotted. Principal components analysis (PCA) and t-distributed stochastic neighbor embedding (t-SNE) were applied to verify the subtype assignments according to the expression profiles of the above genes.

### Establishment of Nomogram

The independent clinical factors validated by univariate and multivariate Cox regression analysis were enrolled to construct a nomogram for prognosis prediction, which included age, stage, and RiskScore. In addition, calibration curves for 3- year prediction were plotted to evaluate the consistency between the actual and predicted survival rates. The closer the curves based on the actual and predicted survival rates are, the better the predictive power of this model.

### Functional Enrichment Analysis

The DEGs between low- and high-risk groups were also identified in TCGA cohort using “limma” package in R software version 4.0.3. Genes with FDR<0.05 and |log FC|>1 was selected for further analysis. The enrichment analysis of Gene Ontology (GO) and Kyoto Encyclopedia of Genes and Genomes (KEGG) were carried out by “clusterProfiler”, “enrichplot”, and “ggplot2” packages in R. GO results included molecular function (MF), biological process (BP), and cellular component (CC). GSEA was performed to explore the potential mechanisms of genes affecting prognosis and obtain the signaling pathways of up-regulation and down-regulation.

### Analysis of Immune Landscape

ImmuneScore, StromalScore, and ESTIMATEScore of each patient were calculated using ESTIMATE algorithm through the “estimate package” in R. The higher ImmuneScore or StromalScore is, the larger amount of the immune or stromal components exits in TME. ESTIMATEScore is the sum of ImmuneScore and StromalScore denoting the overall proportion of immune and stromal components in TME. The fraction of 22 immune cell types for each sample was yielded through cell type identification by estimating relative subsets of RNA transcripts (CIBERSORT; https://cibersort.stanford.edu/). The algorithm of 1,000 permutations was adopted. Only samples with a CIBERSORT *p* of <0.05 were included to perform the subsequent analysis of comparing differential immune infiltration levels between high and low-risk groups (Wu et al., 2020). The differences in TME features including 29 immune functions between the low- and high-risk groups were further explored by “limma”, “GSVA”, “GSEABase”, “ggpubr”, and “reshape2” packages in R.

### Sensitivity of High- and Low-Risk Groups to Immunotherapy and Chemotherapy

“limma” and “reshape2” packages were used to explore the relationship between the RiskScore and the expression level of immunosuppressive molecules. Gene expression data with immunotherapy were downloaded from the GEO database (GSE91061) and analyzed to determine the expression level between responders and non-responders. The expression profiling of 109 melanoma patients and their response to anti-CTLA4 and anti-PDL1 therapy were extracted from GEO: GSE91061 dataset. The sensitivity of each patient to chemotherapy drugs and the IC50 was quantified via the “pRRophetic” package in R. The half-maximal inhibitory concentration (IC50) of six commonly applied chemotherapeutic agents (Cisplatin, Gemcitabine, Vinblastine, Vinorelbine, Paclitaxel, and Sorafenib) on cancer cell lines were obtained from the Genomics of Drug Sensitivity in Cancer database (GDSC, https://www.cancerrxgene.org/). The predictive procedure was conducted with pRRophetic R package. After integrating the gene expression profiles of melanoma tissues, drug IC50 values for melanoma patients were determined by ridge regression analysis of the pRRophetic package, and the prediction accuracy was assessed by 10-fold cross-validation.

### Screening Small-Molecule Drugs

The DEGs between the high- and low-risk groups were uploaded into the CMAP database (https://clue.io/CMAP). Candidate small-molecular drugs were discovered by CMAP mode of action analysis. The enrichment score of each hypothetical drug ranges from −1 to 1. The negative enrichment score of a drug represents its reversal effect on the input DEGs, thus indicating its anti-tumor ability on the cancer-related gene set. % non-null represents the percentage of meaningful results obtained in n experiments conducted in the CMAP database. Small molecule compounds with *p* value < 0.05 and enrichment value < −0.6 were selected.

### Human Protein Atlas Analysis

The immunohistochemistry expression graph of related genes was obtained from the HPA database^4^.

### Patients and Specimens

This study was approved by the Ethics Committee of the Dermatology Hospital of the Chinese Academy of Medical Sciences. Melanoma patients involved were signed informed consent and underwent surgery from June 2019 to September 2020. For expression analysis, a total of 24 pairs of melanoma tissues and corresponding adjacent normal tissues were immediately frozen and stored in liquid nitrogen until RNA extraction. The patient’s baseline characteristics, including age, gender, tumor location, tumor recurrence, and histological subtype, were collected from medical records.

### RNA Extraction and Real-Time Quantitative PCR

Total mRNA was isolated from tissues using Trizol® reagent (Invitrogen). The purity and concentration of RNA were determined by using a NanoDrop 2000 spectrometer (Thermo Fisher Scientific, Waltham, MA, United States). Genomic DNA was eliminated, and reverse transcription reaction was carried out using an Evo M-MLV RT Kit (Accurate Biology, Hunan, China). Expression levels were quantified by qPCR in a LightCycler 480 Instrument II device (Roche Applied Science, Mannheim, Germany) using SYBR Green Premix Pro Taq HS qPCR Kit (Accurate Biology, Hunan, China). All primers were provided by General Biotech Co., Ltd. (Shanghai, China) (Supplementary Table S1).

### Statistical Analysis

Survival analysis was performed through “survminer” and “survival” packages by R software. Kaplan-Meier (K-M) survival curves were drawn to analyze the relationship between RiskScore, tumor mutation burden (TMB) and the OS of melanoma patients. Nonparametric Wilcoxon rank-sum test was used to compare the TMB [TMB = (total count of variants)/(the whole length of exons)] between high-risk group and low-risk group. Correlation between RiskScores and Clinicopathological Characteristics was accomplished by “ggpubr” in R. The statistical significance was tested by Wilcoxon rank-sum test or Kruskal-Wallis rank-sum test. *p* values of <0.05 were regarded as statistically significant.

## Results

### Identification of Ferroptosis-Related Prognostic Gene Signature in0020The Cancer Genome Atlas Cohort

**FIGURE 2:**
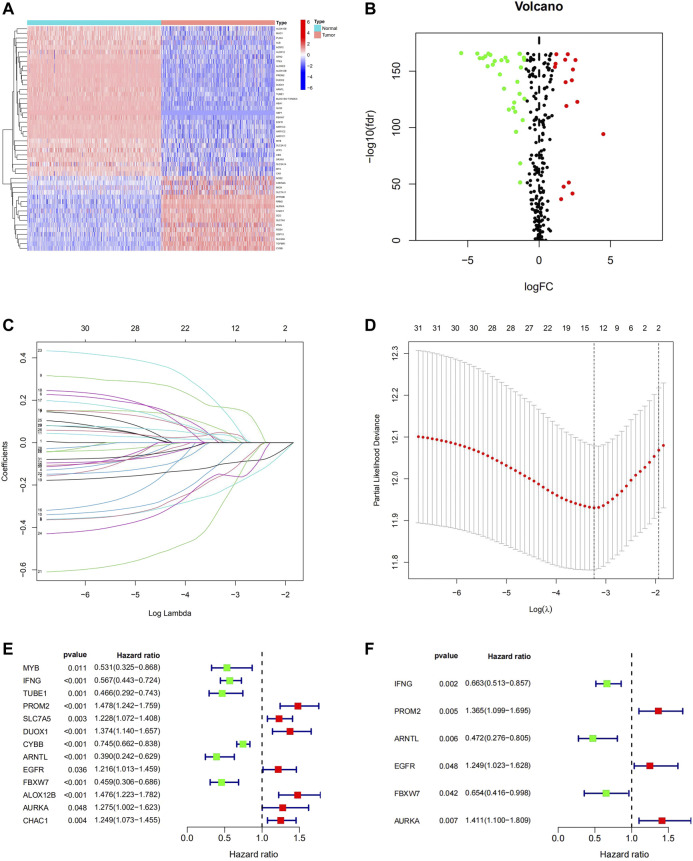
Identification of Ferroptosis-Related Prognostic gene signature in the TCGA Cohort. The 48 DEGs between the normal skin tissues and melanoma tissues in TCGA were displayed in the heatmap **(A)** and volcano map **(B)**. LASSO coefficient spectrum of 48 genes in melanoma **(C)**. Cross-validation for tuning parameter selection in the proportional hazards model **(D)**. 13 prognostic Genes were discovered in univariate cox analysis **(E)** and 6-FRGs signature was identified via multivariate Cox analysis **(F)**.

To profile the expression pattern of FRGs in melanoma, the RNA sequencing (RNA-seq) data of 471 melanoma samples from TCGA-SKCM and 556 normal skin samples from GTEx were combined. We found that the expression of 48 genes, among 259 FRGs, was significantly different between melanoma tissues and normal skin tissues. Of these, 17 genes were up-regulated and 31 genes were down-regulated (Figures 2A,B; Supplementary Table S2). Univariate Cox and LASSO regression analysis were conducted to identify the genes associated with the OS of melanoma patients in the TCGA database (Figures 2C,D), and 13 characteristic genes were determined (Figure 2E). Finally, we performed stepwise multivariate Cox regression analysis and constructed a 6-FRGs signature. The results indicated that high expression of PROM2, EGFR, AURKA, and low expression of IFNG, ARNTL, FBXW7 correlated with a poor prognosis of melanoma patients (Figure 2F).

### Construction and Validation of the Ferroptosis-Related Prognostic Model

**FIGURE 3:**
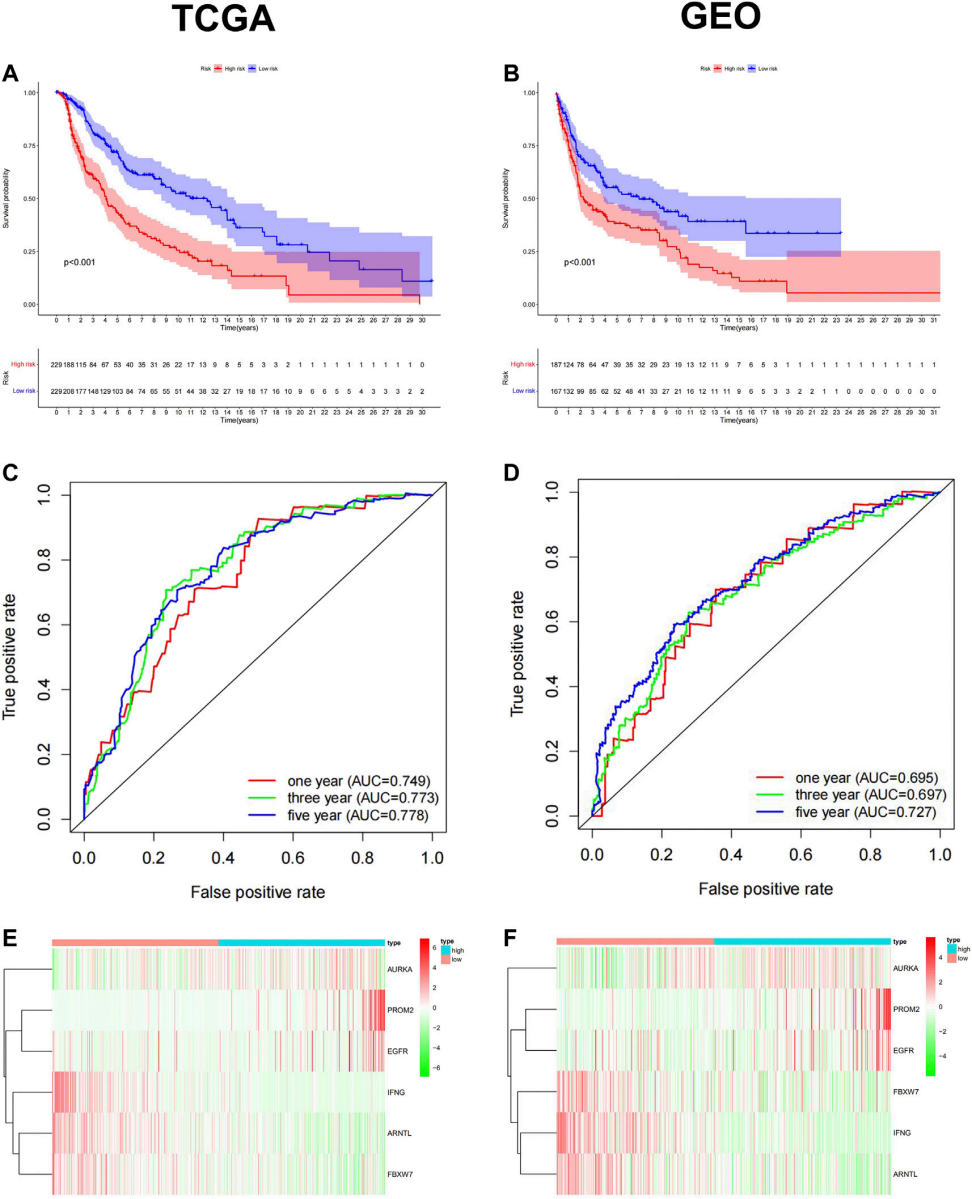
Construction and validation of the ferroptosis-related Prognostic Model in TCGA training dataset and GEO testing dataset. Kaplan-Meier survival curve for OS in the low-risk group and the high-risk group, TCGA **(A)** and GEO **(B)**. The ROC curve confirmed that the high accuracy of the prognostic model in both TCGA**(C)** and GEO**(D)** (TCGA: 1 year AUC = 0.749, 2 years AUC = 0.772, 3 years AUC = 0.773; GEO: 1 year AUC = 0.695, 2 years AUC = 0.747, 3-year AUC = 0.697). Heatmap of the 6-FRGs expression profiles in the prognostic signature of melanoma in TCGA **(E)** and GEO **(F)**.

To ensure the prognostic prediction capability of the 6-FRGs signature. The RiskScore of each patient was calculated, and they were divided into high- and low-risk groups based on the median RiskScore. Finally, a ferroptosis-related prognostic model was constructed in the TCGA cohort. To validate the stability of this prognostic model, prognosis analysis was also performed in combined GEO datasets (GSE15605, GSE19234, GSE22154, GSE54467, and GSE65904). The results in the two cohorts were consistent. In survival analysis, patients in the low-risk group had significantly longer OS than those in the high-risk group (Figures 3A,B, *p* < 0.001). The ROC curve confirmed that the model had high accuracy in predicting OS in both TCGA and GEO (Figures 3C,D; TCGA: 1 year AUC = 0.749, 2 years AUC = 0.772, 3 years AUC = 0.773; GEO: 1 year AUC = 0.695, 2 years AUC = 0.747, 3-year AUC = 0.697). The growing expression of AURKA, PROM2, EGFR was observed with a RiskScore increase in the risk heatmap, whereas IFNG, ARNTL, FBXW7 behaved oppositely (Figures 3E,F). The distribution of RiskScore and survival time in the TCGA training dataset and GEO testing dataset, respectively (Supplementary Figures S1A–D). Likewise, PCA and t-SNE determined that two risk groups were distinguished in a discrete direction (Supplementary Figures S1E–H). These results demonstrated that RiskScore was a robust prognostic predictor in melanoma clinics.

### The Prognostic Capacity of the 6-Ferroptosis-Related Genes Signature

**FIGURE 4:**
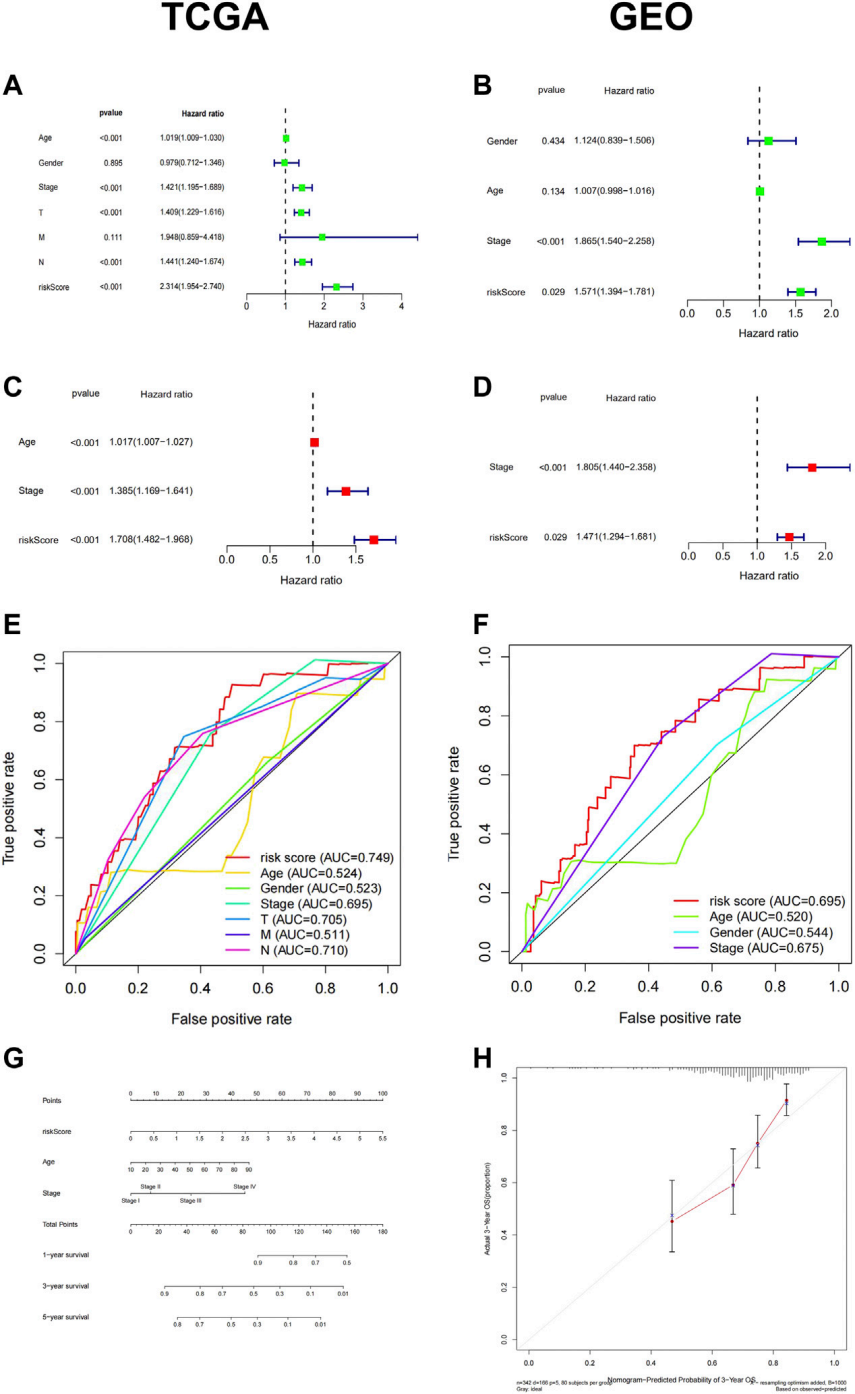
Cox regression and a nomogram of the signature of 6-FRGs predicting survival in melanoma patients. Univariate Cox regression analysis and multiple Cox regression analysis confirmed RiskScore could predict the prognosis of patients both in TCGA **(A,C)** and GEO **(B,D)** independently. The ROC curve showed the high prediction accuracy of this model in TCGA **(E)** and GEO **(F)**. **(G)** A nomogram of the 6-FRGs signature for 1, 3, 5-year survival prediction in the TCGA dataset. **(H)** The 3-year OS calibration curve showed good calibration in the training set.

Subsequently, we conducted univariate and multivariate Cox analyses to assess whether the signature RiskScore was an independent prognostic factor for patient survival. Available clinical variables were obtained and we demonstrated that the 6-FRGs signature could predict the prognosis of patients independently both in TCGA and GEO cohort (Figures 4A–D) (univariate cox: TCGA cohort: HR = 1.746, 95% CI = (1.515, 2.012), *p* < 0.001; GEO cohort: HR = 1.571, 95% CI = (1.394, 1.781), *p* = 0.029; multivariate cox: TCGA cohort: HR = 1.708, 95% CI = (1.482, 1.968), *p* < 0.001; GEO cohort: HR = 1.471, 95% CI = (1.294, 1.681), *p* = 0.029). Meanwhile, the 6-FRGs signature exceeded the prediction accuracy for any single clinical variable, such as age, TMN, and stage classification (Figures 4E,F). In order to comprehensively evaluate the prognosis of melanoma patients with clinical information, we constructed a nomograph based on the results of the multivariate analyses. Three prognostic markers were included to predict the survival rates of melanoma patients at 1, 3, and 5 years including RiskScore, age and stage (Figure 4G). The calibration curve for the OS showed an optimal agreement between the prediction by the nomogram and actual observation (Figure 4H). In addition, there was a significant correlation between RiskScore and T staging in TCGA, but not with other clinical information (Supplementary Figures S2A–F). The highest increase in RiskScore was found in patients at the T4 grade in TCGA, likewise, there were significantly more T4 patients in the high-risk group (Supplementary Figures S2G–H). In general, the 6-FRGs signature might serve as potential biomarkers and prognostic factors for melanoma.

### Ferroptosis Related Risk Score is Associated With Tumor Mutation Burden Features in Melanoma

**FIGURE 5:**
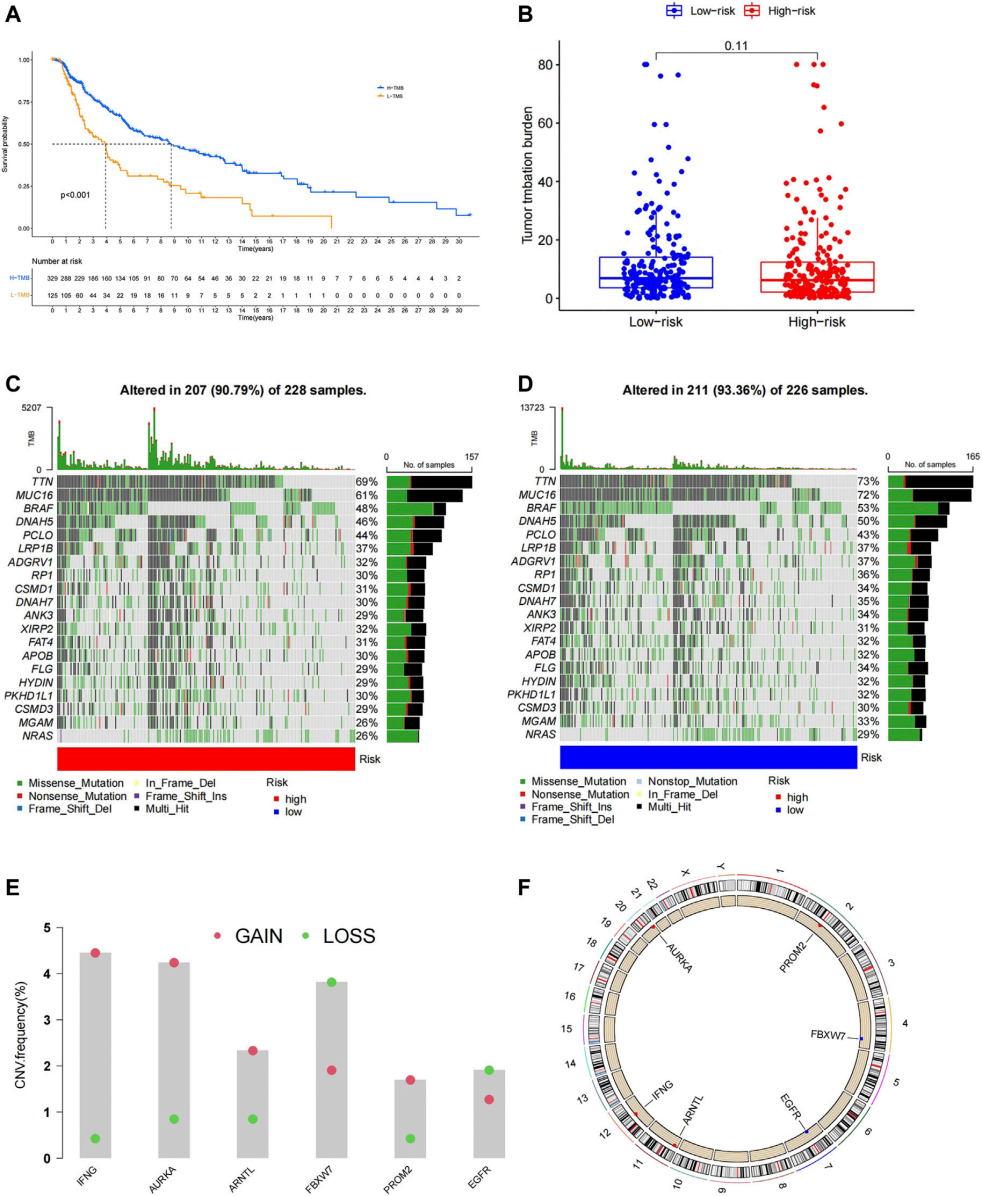
**(A)**Patients with high TMB had a longer OS. **(B)**No significant difference in total TMB between the high- and low-risk group. **(C,D)** High-frequency mutated genes are ranked based on the mutation frequency, patients in the low-risk group showed higher mutation frequency. **(E)** Analysis of CNVs, the six prognostic FRGs exhibit different degrees of gain and loss in CNVs in melanoma. **(F)** Position of the six model genes on chromosome.

The TMB is related to the prognosis and response to immunotherapy in specific cancer types. Here, we found that melanoma patients with higher TMB showed better OS (Figure 5A). We analyzed the top 20 high-frequency mutated genes in melanoma in the TCGA database and found that the low-risk group exhibited a more frequent mutation rate than the high-risk group (Figures 5C,D). However, no significant difference in total TMB was found in the two groups (Figure 5B). We further explored the CNVs of the six FRGs in the TCGA database. Somatic copy number alterations were shown as bar plots with copy number gain in red and loss in green (Figure 5E). The chromosomal location of the six FRGs was marked in Figure 5F.

### Functional Annotations of the 6-Ferroptosis-Related Genes Signature

**FIGURE 6:**
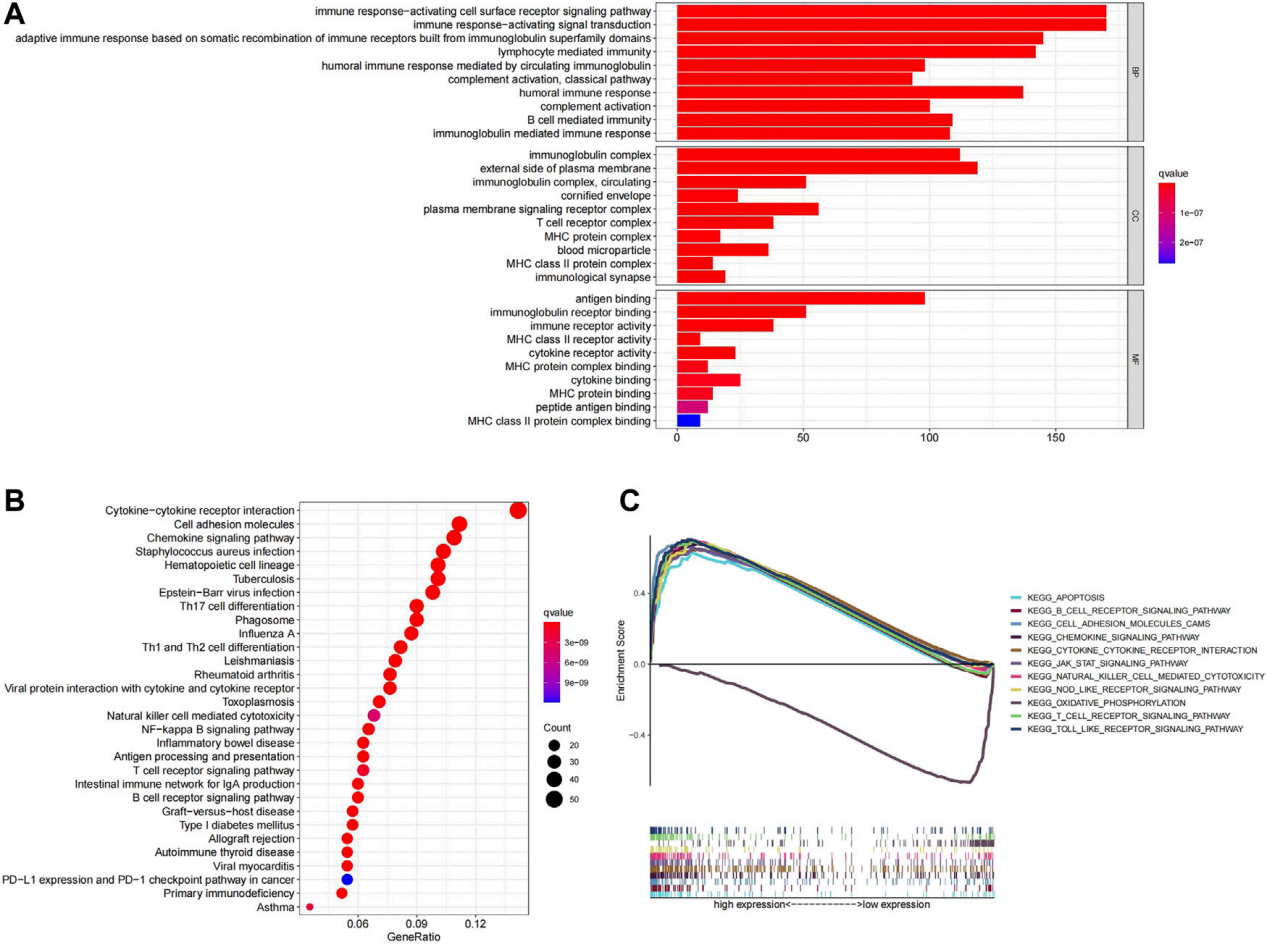
**(A)**Top ten GO terms were selected for visualization. **(B)**Twenty KEGG pathways were visualized. **(C)**GSEA analysis, the differences in enrichment pathways between high- and low-risk groups were shown.

To elucidate the biological functions and pathways related to the prognostic model, a total of 904 DEGs between the high- and low-risk groups in TCGA were identified and used for GO enrichment and KEGG pathway analysis. GO terms associated with immune functions were significantly enriched, such as immune response−activating cell surface receptor signaling pathway, immune response−activating signal transduction (Figure 6A). KEGG enrichment analysis also demonstrated a high enrichment of immune pathways, including the T/B cell receptor signaling pathway, cytokine signaling pathway, and PD-1 checkpoint pathway in cancer (Figure 6B). Similarly, GSEA analysis indicated that multiple immune-related pathways were activated in the low-risk group, including B cell receptor signaling pathway, Natural killer cell mediated cytokine, JAK signaling pathway, etc. (Figure 6C).

### 6-Ferroptosis-Related Genes Signature is Highly Correlated With Immune Function and Immune Checkpoints in Melanoma

**FIGURE 7:**
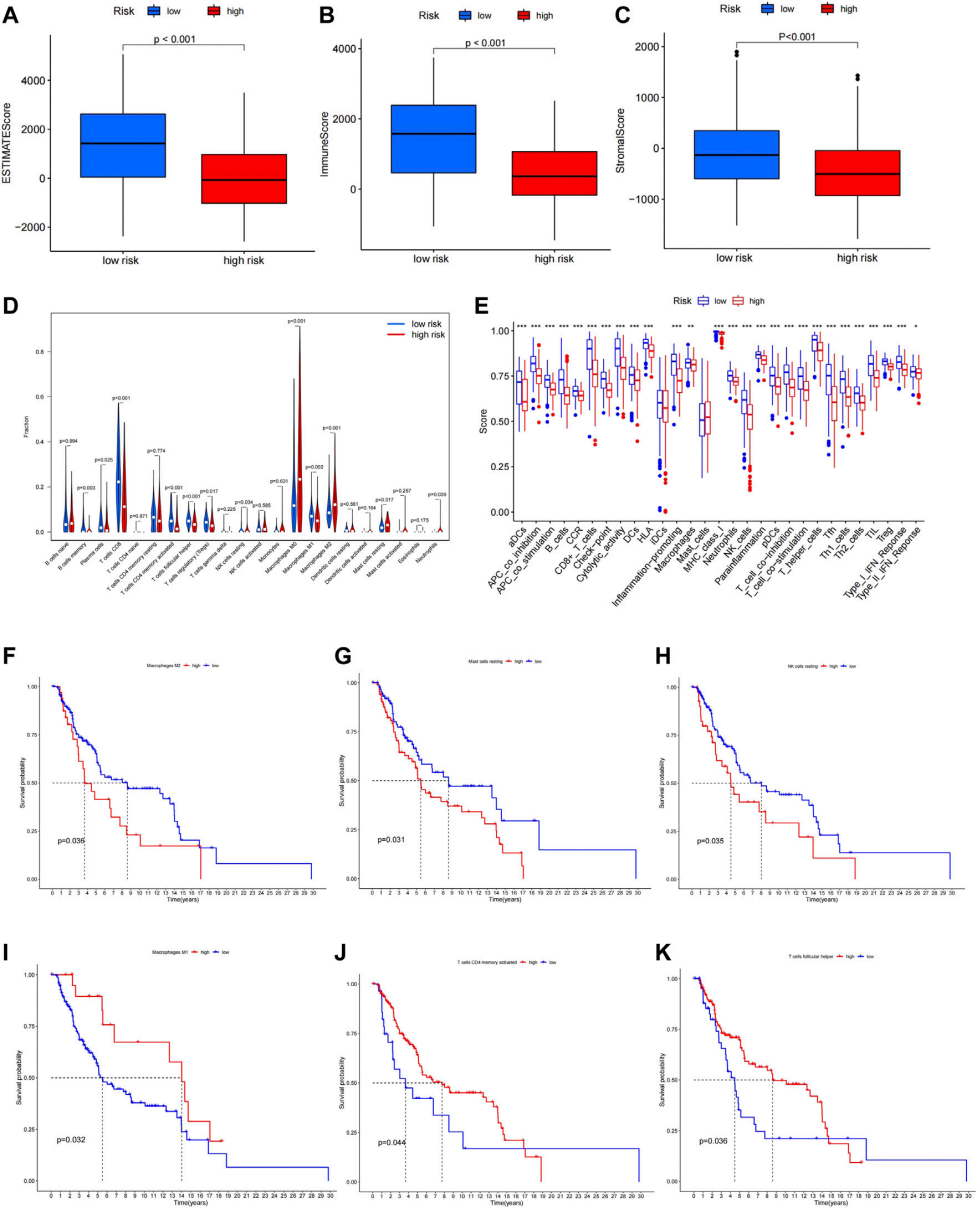
ESTIMATEScore **(A)**, ImmuneScore **(B)**, and StromalScore **(C)** in high- and low-risk group. **(D)** Immune infiltration levels of 22 immune cell types in the high- and low-risk group. **(E)**Multiple immune-related functions in two groups. High infiltration of Macrophages M1, T cells CD4 memory activated, and T cells follicular helper was related to longer OS **(F–H)**, while high infiltration of Macrophages M2, NK cells resting, and Mast cells were related to shorter OS **(I–K)**.

The function enrichment analysis suggested a close correlation between ferroptosis and immune function in melanoma, thus, we investigated the relationship between our prognostic model and immune status of melanoma patients. We found that the ESTIMATEScore, ImmuneScore, and StromalScore were significantly higher in the low-risk group compared to the high-risk group (Figures 7A–C). In Immune cell infiltration analysis, significant differences in infiltrating immune cell types were found between the high- and low-risk group (Figure 7D). After combining the results of the survival analysis, Macrophages M1, T cells CD4 memory activated, and T cells follicular helper with high infiltration in the low-risk group were all related to a better prognosis (Figures 7F–H), while Macrophages M2, NK cells resting, and Mast cells resting with high infiltration in the high-risk group were related to worse prognosis in melanoma (Figures 7I–K). The differences in immune function between the high- and low-risk groups were also explored. The results demonstrated that most immune-related functions were upregulated in the low-risk group, which were associated with better prognosis (Supplementary Figure S3). The results above indicated that the alternations in immune status might be one of the reasons for ferroptosis regulating the progression of melanoma.

### Efficiency Prediction of Prognostic Model for Immunotherapy Response

**FIGURE 8:**
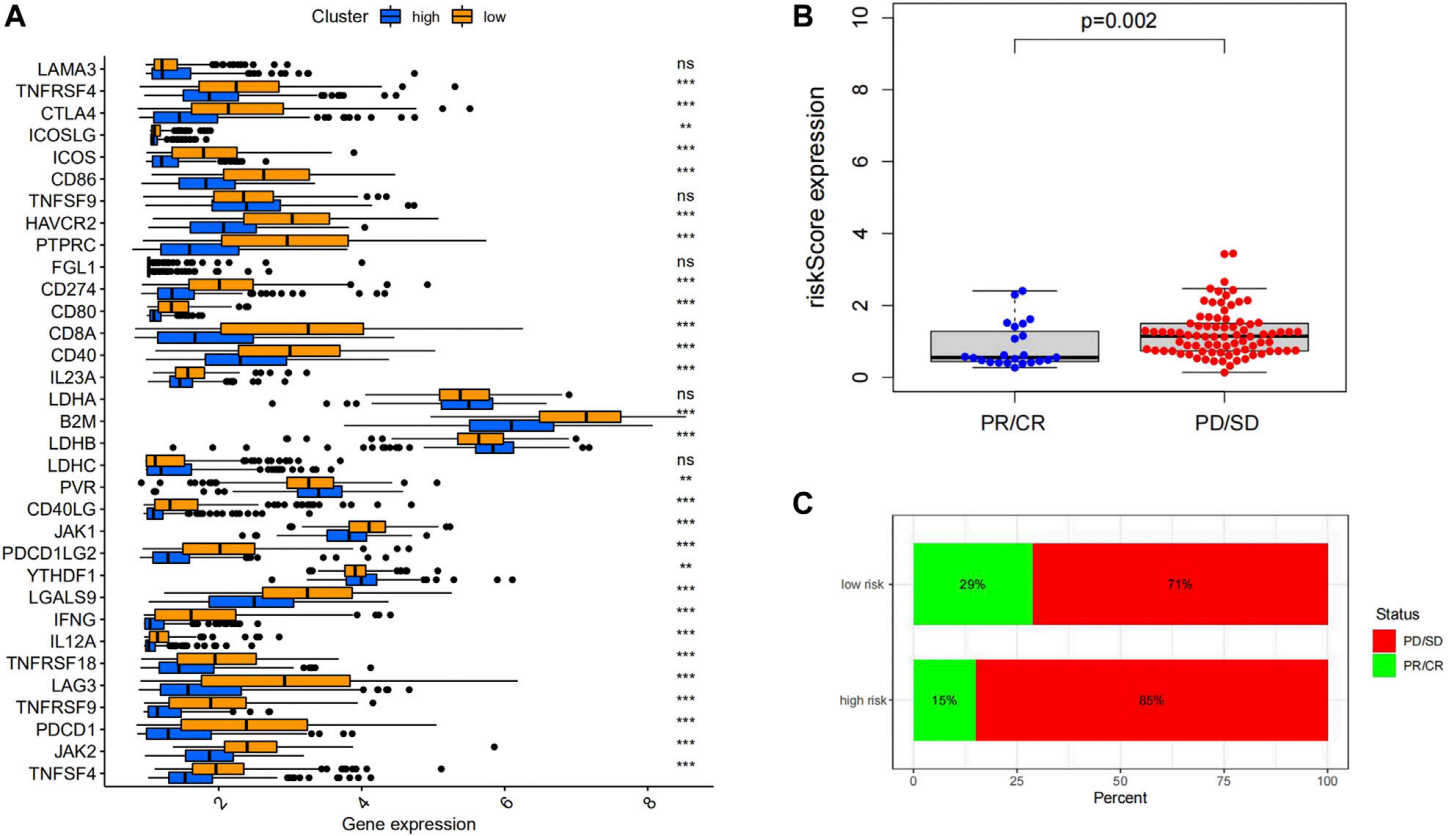
**(A)** ICPs related genes were upregulated in the low-risk group. **(B)** Immunotherapy responders had significantly lower RiskScores than non-responders. **(C)** The proportion of responders in the low-risk group was higher than high-risk group. **(D)** The distribution of 22 immune cells was different between the high- and low-risk groups.

We compared the differences in the immunotherapy response rate of the high- and low-risk groups. The results showed that most of the immune checkpoint molecules were highly expressed in the low-risk group (Figure 8A), suggesting better response to immunotherapy in patients of the low-risk group. This conclusion was further confirmed using clinical data from the GEO database (GSE91061): the distribution of immunotherapy responders for high-, and low-risk groups was 15 and 29%, respectively. Thus, a significant lower response rate was observed in high-risk group (Figures 8B,C). The prognostic model might contribute to the future screening of patients who can benefit from immunotherapy. In addition, we found CD8^+^ T cells and naive B cells infiltrated were significantly upregulated in responders (Supplementary Figure S4), indicating they might play an important role in the response to immunotherapy.

### Identification of Sensitivity to Chemotherapeutic and Drug Screening for Two Groups

**FIGURE 9:**
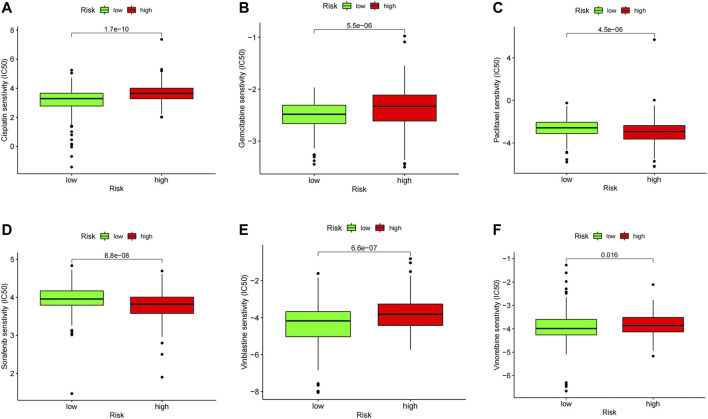
IC50 values of several commonly used chemotherapeutic drugs in the high- and low-risk group including Cisplatin **(A)**, Gemcitabine **(B)**, Vinblastine **(C)**, and Vinorelbine **(D)**, Paclitaxel **(E)** and Sorafenib **(F)**.

To further explore the possibility of applying our prognostic model in melanoma therapy, we assessed differences in drug sensitivity between the high- and low-risk group by analyzing the IC50 of chemotherapeutic agents. The results indicated that patients in the high-risk group were more sensitive to Cisplatin, Gemcitabine, Vinblastine, and Vinorelbine, while patients in the high-risk group were more sensitive to Paclitaxel and Sorafenib (Figures 9A–F). Furthermore, through the CMAP database, 10 potential small molecule drugs were screened out, which might be potential therapeutic targets for high-risk populations (Table 1), of which phenazone was the most likely to be involved in tumor immunity through ferroptosis.

**TABLE 1:** Ferroptosis-related potential small-molecules for melanoma therapy.

CMap name	*n*	Enrichment	*p*-value	Percent non-null
pilocarpine	4	−0.813	0.00229	50
ambroxol	4	−0.782	0.00454	50
phenazone	3	−0.837	0.00859	66
cicloheximide	4	−0.721	0.01239	75
emetine	4	−0.68	0.02304	75
erythromycin	5	−0.601	0.029	60
antazoline	4	−0.656	0.03219	75
amphotericin B	4	−0.649	0.03557	50
Prestwick-984	4	−0.641	0.03981	50
oxybenzone	4	−0.636	0.04269	50
dexibuprofen	4	−0.632	0.0444	50
hecogenin	4	−0.632	0.0446	50

### The Expression of six Ferroptosis-Related Genes in Human Protein Atlas and Chinese Melanoma Tissues

**FIGURE 10:**
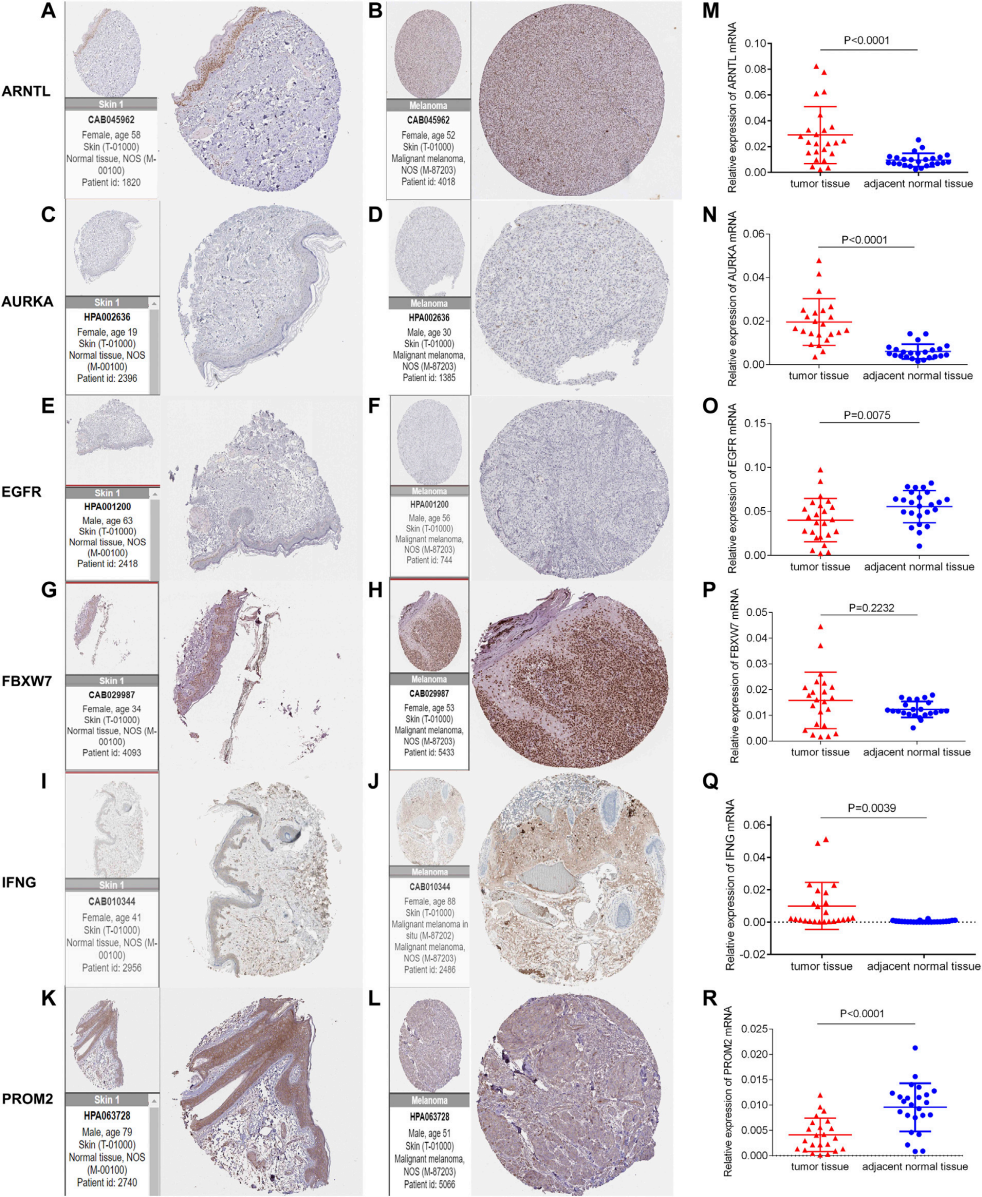
Immunohistochemistry of six genes in normal skin tissues and melanoma tissues (A-L). The mRNA expression level of six genes in 24 pairs of melanoma tumor and adjacent normal tissues was measured by qRT-PCR (M-R).

The protein expression of six genes in normal skin and melanoma tissues was compared through the immunohistochemistry obtained from HPA (Figures 10A–L). The mRNA level of ARNTL, AURKA, and IFNG in melanoma tissues were significantly higher than those in adjacent normal tissues (Figures 10M,N,Q). While the EGFR, PROM2 mRNA in melanoma tissue was lowly expressed than that in adjacent normal tissues (Figures 10O,R). The mRNA expression of FBXW7 had no significant difference between melanoma tissues and adjacent normal tissues (Figure 10P).

## Discussion

Herein, we identified a novel prognostic model of melanoma patients with 6-FRGs signature in an external cohort and explored the correlation between the signature and clinicopathological features. Not only the six FRGs verified in our study were, respectively, recognized as an independent prognostic factor for melanoma, but such a prognostic model we built here also allowed for better assessment of prognosis in melanoma patients. Functional enrichment and immune landscape revealed that the ferroptosis-related model engaged in the immune process.

The prognostic model comprises six FRGs, including IFNG, ARNTL, FBXW7, PROM2, EGFR, and AURKA. It has been noted that carcinogenesis and the efficacy of anti-tumor therapy are closely associated with the model genes. Firstly, IFNG and EGFR were considered to play a dual role in tumor progression and oncotherapy. In consensus, IFNG enhanced human immune function, protected normal cells from infection and tumor transformation (Zuo et al., 2019). But an interesting phenomenon was that IFNG could promoted T cell exhaustion via the PDL1 pathway in the tumor immune process (Mo et al., 2018; Salerno et al., 2019; Thiem et al., 2019). The antagonism relationship between interferon signaling in cancer cells and immune cells might be the reason explaining the contradictory roles of IFNG in tumor immune response (Benci et al., 2019). EGFR inhibitor has been widely used to treat a variety of tumors (Pecci et al., 2021). Notably, high expression of EGFR results in a slow-growth phenotype with melanoma cells (Sun et al., 2014), but EGFR turned into a cancer-promoting factor and contributed to the targeted therapy resistance to BRAF or MEK inhibitors (Quadri et al., 2021). The bidirectional role in regulating ferroptosis of EGFR might be one of the significant reasons. Blocking the EGFR/MAPK signaling could protect cancer cells from ferroptosis (Poursaitidis et al., 2017). Inversely, EGFR degradation or inactivation leads to the ferroptosis of tumor cells via activating NRF2 (Sun et al., 2021). In our study, elevated EGFR levels are related to a poor prognosis of melanoma patients. FBXW7 is a critical tumor suppressor and one of the most deregulated ubiquitin-proteasome system proteins in human cancer, but its role in melanoma remains (largely) unknown. Limited research revealed its tumor-suppressive function in melanoma (Gstalder et al., 2020) was consistent with our findings. FBXW7, as the ferroptosis driver, might be the potential mechanism in melanoma progression. The prognostic role of Prominin 2 (PROM2) in human cancers is controversial. Mechanistically, reduced iron concentration in cells caused by PROM2 could protect tumors from ferroptosis, and the inhibition of PROM2 transcription sensitizes drug-resistant cancer cells to ferroptosis inducers (Brown et al., 2021). Studies on AURKA and ARNTL in melanoma remain limited. AURKA kinase is an essential serine/threonine kinase for mitosis and chromosome stability. The aberrant amplification and high expression of AURKA in melanoma patients were observed both in the research of Yan et al. and ours (Yan et al., 2018). Another finding was that inhibiting AURKA impaired melanoma growth and survival regardless of whether the melanoma cells are resistant to BRAF/MEK inhibitors (Puig-Butille et al., 2017). The autophagy degradation of ARNTL, a circadian clock-related gene (Li et al., 2021), promotes ferroptosis (Liu et al., 2019) and is related to anti-tumor immunity in metastatic melanoma (de Assis et al., 2018). In general, as crucial components involved in tumor development or inducing treatment resistance, these six FRGs have considerable potential as therapeutic targets or biomarkers in melanoma clinics.

There is an inseparable relationship between ferroptosis and tumor immunity. On the one hand, ferroptosis cancer cells release multiple signaling molecules to reshape tumor immune response, named the “find-me” and “eat-me” immunostimulatory signals. These signaling molecules drive DCs, Macrophages, and other immune cells to localize dead cells correctly (Xu et al., 2021). Meanwhile, activated CD8 + T cells release IFNG to activate the JAK-STAT1 pathway in tumor cells to inhibit the expression of system Xc -, and make tumor cells sensitive to pro-apoptotic stimuli, thereby exerting anti-tumor effects (Lane et al., 2018). On the other hand, tumor cells may also increase immunosuppressive signals under the pro-apoptotic stimulation of IFNG, thereby inhibiting the survival of T cells and promoting the infiltration of immunosuppressive immune cells. Therefore, IFNG-induced ferroptosis is a double-edged sword for tumor development. When the balance shifts to ferroptosis-mediated immunosuppression, the ferroptosis of tumor cells induced by IFNG may weaken anti-tumor immunity and even lead to acquired tolerance to immunotherapy (Shi et al., 2021). Besides, different ferroptotic immune cells play different roles in tumor immunity: ferroptosis of anti-tumor immune cells (including CD8 + T cells, NK cells, and DC) leads to low immune function; ferroptosis of suppressive immune cells, such as M2 tumor-associated macrophages and Tregs, reverse their tumor-promoting functions (Talty and Bosenberg, 2021). In general, existing findings suggest that complex ferroptosis-based crosstalk between tumor cells and immune cells has a two-way regulation effect on cancer development and treatment outcome. In our study, the high-risk group has a higher proportion of M2 type macrophages, NK resting cells, and Mast resting cells, all of which are associated with a worse prognosis; while the low-risk group has a higher proportion of Macrophages M1, T cells CD4 memory activated, and T cells follicular helper, all the three types of cells were associated with a better prognosis in melanoma patients. In addition, a higher risk score is related to impaired anti-tumor immunity. Ferroptosis in tumor cells induced by activated CD8 + T cells (Wang et al., 2019) and the weakened anti-tumor immunity of high-risk patients might be the reason to explain the better prognosis and better effect on immunotherapy in the low-risk group.

Ferroptosis has become of great significance in cancer therapy, promising strategies targeting ferroptosis mainly reflected in the following aspects: ferroptosis modulate tumor sensitization to anti-cancer therapies; ferroptosis-associated anti-tumor combination therapy; ferroptotic small molecules and ferroptosis Nanoparticle inducers of in cancer. Current evidence suggests that tumor microenvironment (TME) is one of the decisive factors that determine whether ferroptosis inducers can improve the efficacy of immunotherapy (Xu et al., 2021). In human cancers, TME was divided into three immunophenotypes: inflamed type, immune-altered type, and immune-desert type. In the inflamed type, ferroptosis inducers could reduce the efficacy of immunotherapy by significantly killing infiltrated CD8 + and CD4 + T cells, weakening normal DC functions, and disturbing the maturation of naive DCs. In the immune-altered type, ferroptosis reverses immunosuppressive TME and immunotherapy resistance by repolarizing tumor-associated macrophages into M1 cells. Radiotherapy and chemotherapy or targeted drugs may be more effective than immunotherapy in the immune-desert type. Combining Chemotherapeutic drugs and ICPs inhibitors might lead to increased tumor immunogenicity and immune cell infiltration by triggering ferroptosis of tumor cells (J. Du et al., 2021; Ren et al., 2021), and in this way, improving the efficacy of immunotherapy (Xu et al., 2021). Some ferroptosis related small-molecules, such as statins, have also been valued in anti-tumor combination drugs (Tang et al., 2020). Overall, a variety of ferroptosis inducers could enhance the efficacy of immunotherapy, and the combined application of them has become a new trend (Tang et al., 2020; Emmons and Smalley, 2021). In our prognostic model, both groups exhibited different sensitivity to chemotherapy drugs. The response rate of anti-PD-1 or anti-CTLA4 immunotherapy in melanoma patients was significantly higher in the low-risk group, while patients in the high-risk group presented a greater sensitivity to chemotherapy. In conclusion, combined with the findings in the immune landscape of the two groups, the TME of the patients in the high-risk group are more likely to be the immune-desert type, while the treatment response of the low-risk group may be similar with the inflamed type in the clinic. Ferroptosis reshaping tumor immune response might be the potential mechanism responsible for this phenomenon. Ferroptosis-related small molecules were selected based on the DEGs between the high and low-risk groups, we speculated their therapeutic value in melanoma, but the underlying mechanism needs further investigation.

In summary, our study defines a new prognostic model for FRGs in melanoma. In the derivation and validation cohort, the model was an independent predictor of OS, providing insights into the prognosis prediction of melanoma patients. More prospective real-world data is needed to verify its clinical utility. Moreover, our research further demonstrated that ferroptosis was strongly correlated with tumor immunity and tumor response to treatment. Targeting the 6-FRGs or small molecules selected above holds great interest as a potential component of combination tumor therapy.

## Conclusion

The present study identified a novel FRG signature for prognostic prediction in melanoma. Based on the signature-related immune infiltration landscape found in our study, targeting the FRGs might be a therapeutic alternative for melanoma.

## Data Availability

The original contributions presented in the study are included in the article/Supplementary Material, further inquiries can be directed to the corresponding authors.

## Footnotes

1
https://portal.gdc.cancer.gov/.

2 https://genome.ucsc.edu/gtex.html.

3
https://www.ncbi.nlm.nih.gov/geo/.

4
https://www.proteinatlas.org/.

## Abbreviations

FRGs, Ferroptosis-Related Genes; TCGA, The Cancer Genome Atlas; GEO, Gene Expression Synthesis; HPA, Human Protein Atlas; qRT-PCR, quantificational real-time polymerase chain reaction; OS, Overall Survival; ROC curve, Receiver operating characteristic curve; TICs, Tumor-infiltrating Immune Cells; ICPs, Immune checkpoints; TNM, Tumor-lymph Node-metastasis; DEGs, differentially expressed genes; log_2_FC, log_2_ fold change; FDR, false discovery rate; AUC, area under curve; PCA, principal components analysis; t-SNE, t-distributed stochastic neighbor embedding; GO, Gene Ontology; KEGG, Kyoto Encyclopedia of Genes and Genomes; MF, molecular function; BP, biological process; CC, cellular component; TMB, tumor mutation burden; CNVs, copy number variations; GSEA, Gene Set Enrichment Analysis; CMAP, Connectivity Map; TME, tumor microenvironment.
