# Supplementary material for: Construction and Validation of a 6-Ferroptosis Related Gene Signature for Prognosis and Immune Landscape Prediction in Melanoma
Source: Front Genet. 2022 May 25;13:887542. doi: 10.3389/fgene.2022.887542 (PMC9174666; doi:10.3389/fgene.2022.887542)
Supplement: Supplementary file 3 [file DataSheet1.PDF]

Supplementary Material

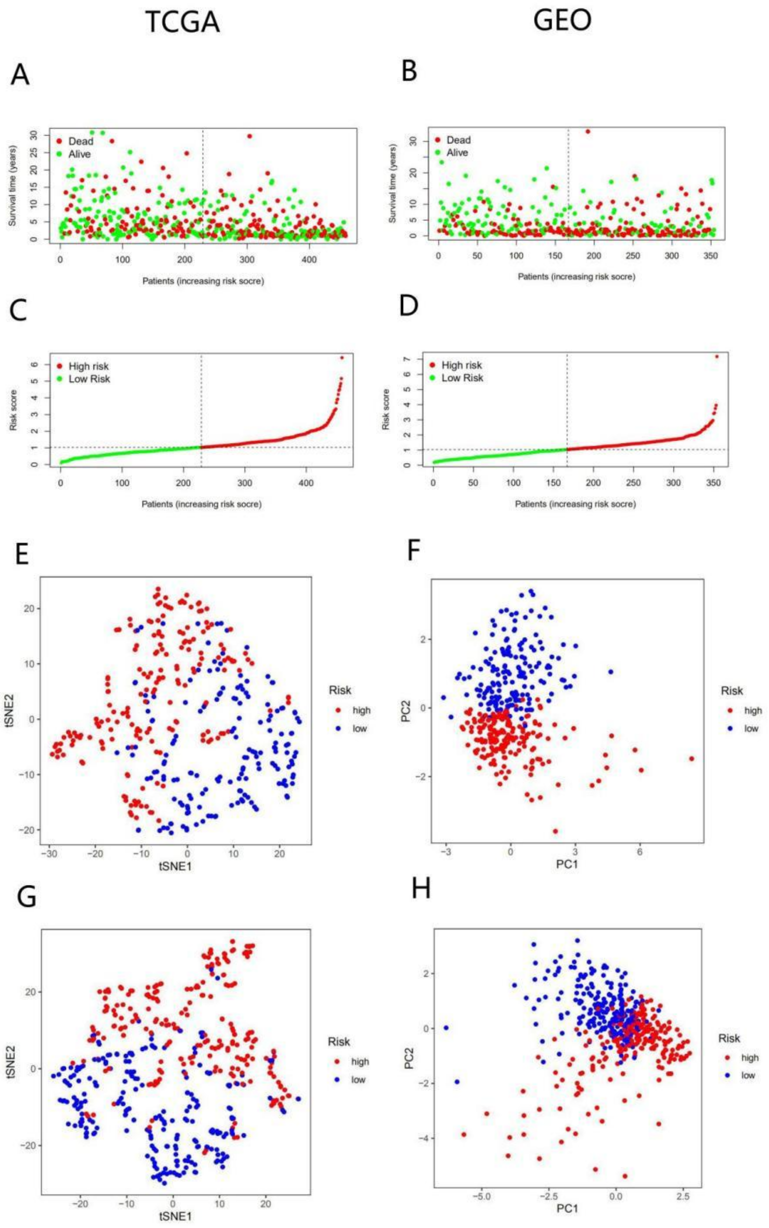

Supplementary Figure1. The distribution of survival time (A, B) and RiskScore (C,D) in the TCGA training dataset and GEO testing dataset. PCA (E-F) and t-SNE (G-H) results indicated the risk model could distinguish high- and low- risk model well in the TCGA training dataset and GEO testing dataset.

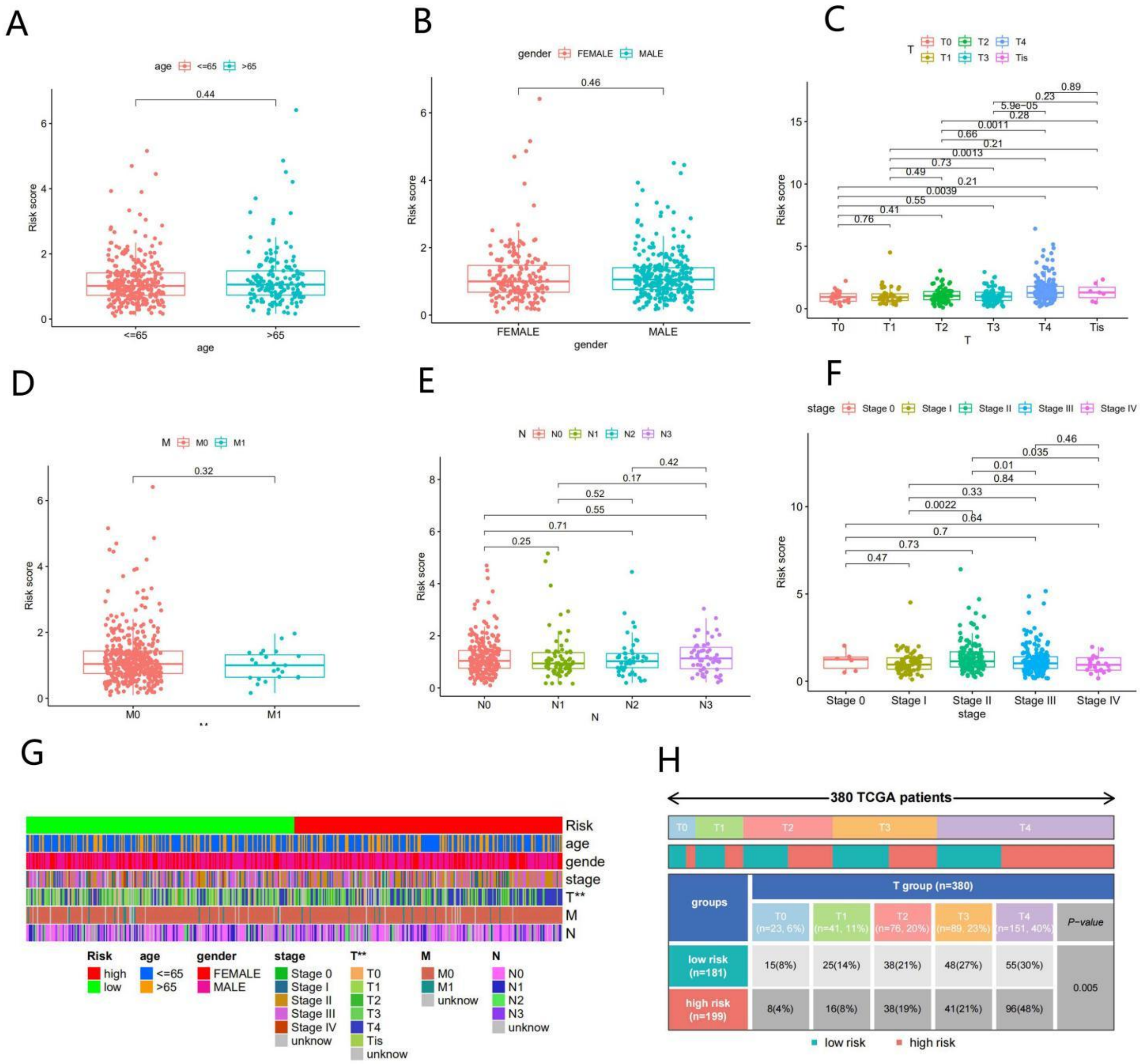

Supplementary Figure 2. Scatter plots showed that RiskScore was positively correlated with T staging in TCGA (C), but not with others clinical variable (A, B, D, E, F). The RiskScore was significantly higher in patients at the T4 (F) and meanwhile, more patients were during T4 stage among the high-risk group than low-risk group (G-H).

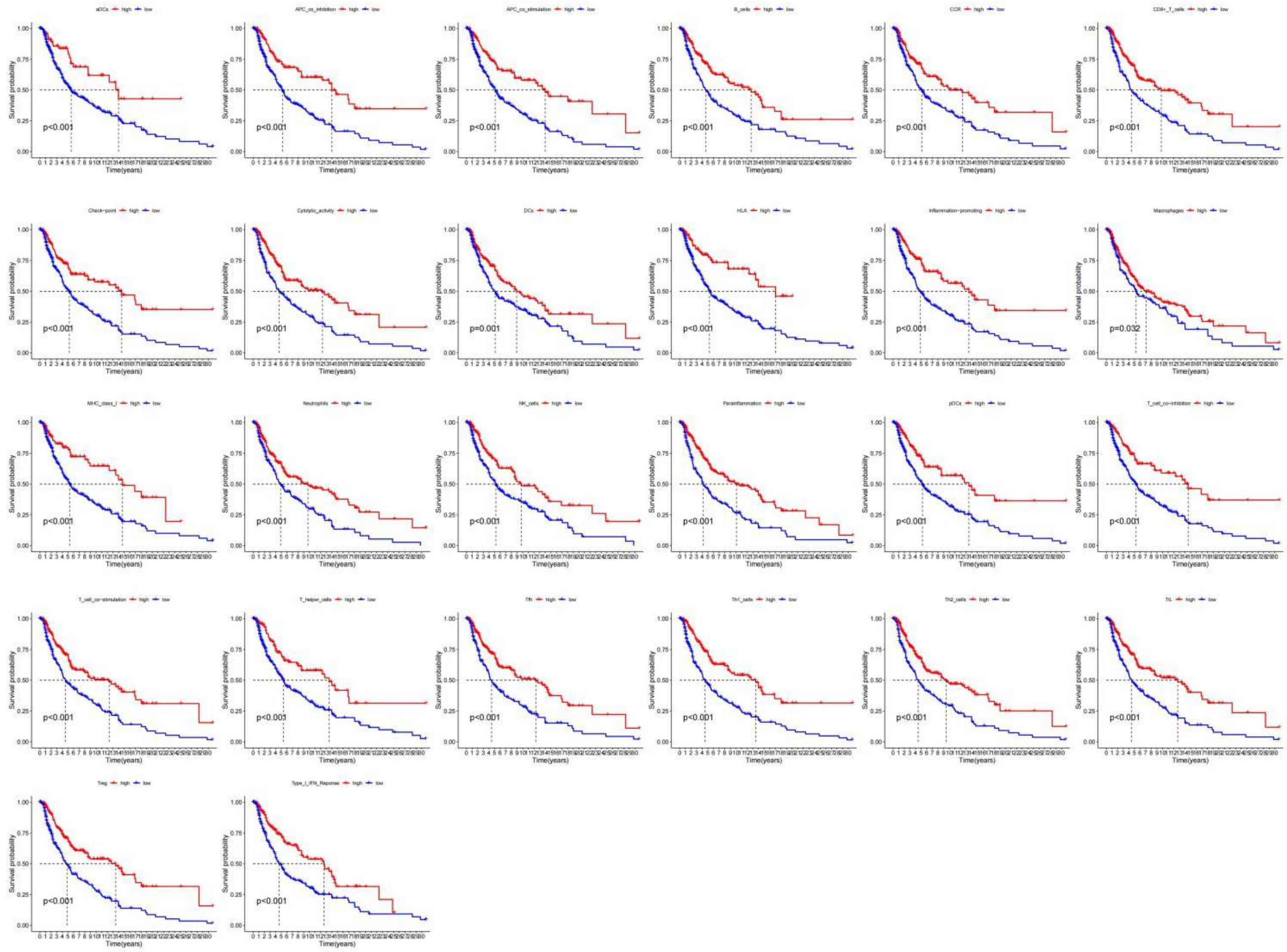

Supplementary Figure3. 26 out of 28 kinds of immune-related functions were up-regulated in the low-risk group, including functions of aDCs, APC co inhibition, APC co stimulation, B cells, CCR, CD8+ T cells, Check-point, Cytolytic activity, DCs, HLA, Inflammation-promoting, Macrophages, MHC class I, Neutrophils, NK cells, Parainflammation, pDCs, T cell co-inhibition, T cell co-stimulation, T helper cells, Tfh, Th1 cells, Th2 cells, TIL, Treg, Type I IFN Reponse, all of which were associated with better prognosis in melanoma.

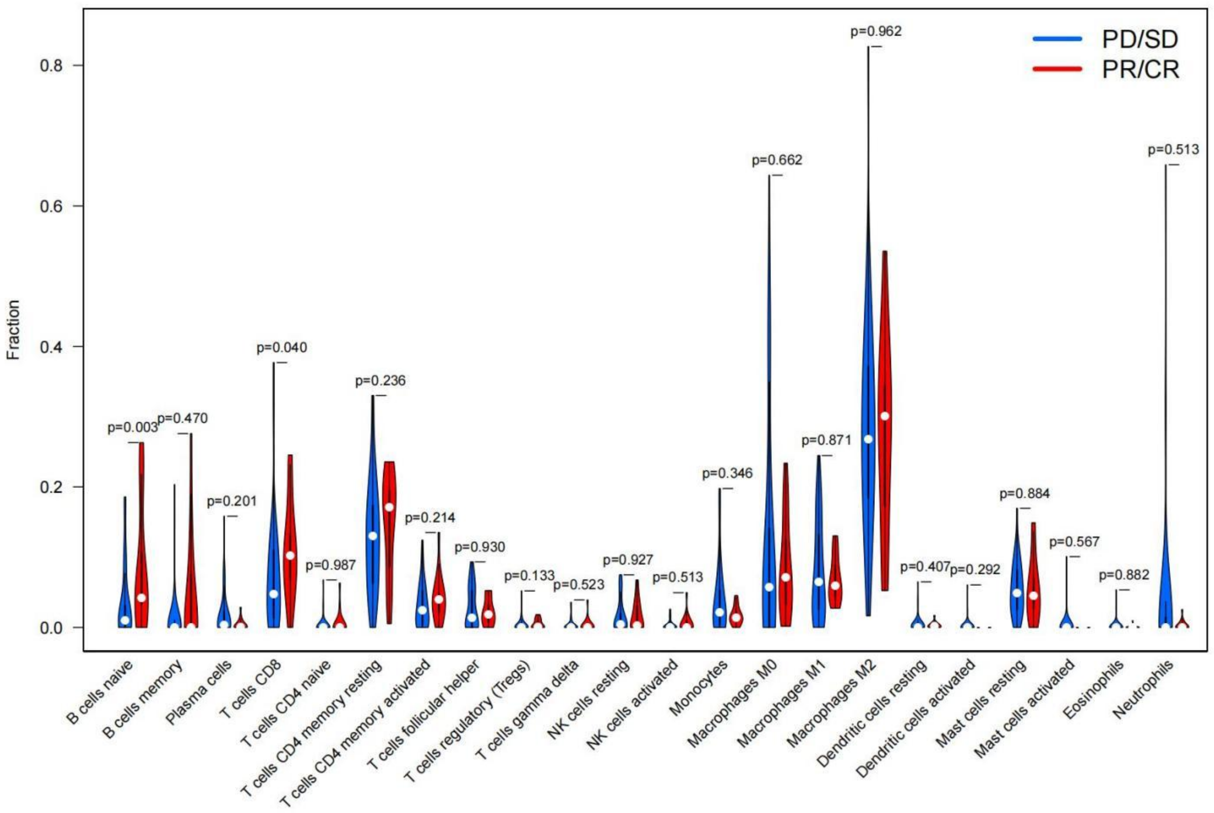

Supplementary Figure 4. CD8+ T cells and naive B cells infiltration were significantly upregulated in responders.

Table S1. Primers used in this study

**Gene**	**Primer sequence (5'-3')**	
**qRT-PCR**		
IFNG	F: TCGGTAACTGACTTGAATGTCCA	R: TCGCTTCCCTGTTTTAGCTGC
EGFR	F: TTGCCGCAAAGTGTGTAACG	R: GTCACCCCTAAATGCCACCG
FBXW7	F: CGACGCCGAATTACATCTGTC	R: CGTTGAAACTGGGGTTCTATCA
ARNTL	F: AAGGGAAGCTCACAGTCAGAT	R: GGACATTGCGTTGCATGTTGG
PROM2	F: GGGCCACAGACTGCAAGTT	R: AGCTCATTCAGTAGGGCCTTTA
AURKA	F: GGAATATGCACCACTTGGAACA	R: TAAGACAGGGCATTTGCCAAT
GAPDH	F: GAGTCAACGGATTTGGTCGT	R: TTGATTTTGGAGGGATCTCG

Table S2. Ferroptosis-related DEGs between normal skin tissues and melanoma tissues

gene	Normal Mean	Melanoma Mean	Log FC	p-Value	fdr
SLC7A11	0.355117489	1.167470157	1.717017356	1.61E-48	2.46E-48
AKR1C1	4.410133532	0.413756299	-3.413969158	2.27E-166	4.03E-165
AKR1C2	4.338504016	0.368989995	-3.555544059	5.85E-168	2.70E-166
AKR1C3	3.803589625	0.816986304	-2.218977802	1.67E-160	1.11E-159
MUC1	3.190272708	1.236493623	-1.367424953	3.71E-131	1.10E-130
SCD	3.417963018	7.351537305	1.104909181	2.94E-155	1.62E-154
TP63	5.537046081	0.642117777	-3.108206684	1.24E-160	8.71E-160
CBS	1.702115142	0.252985538	-2.750201816	1.86E-148	7.54E-148
ARNTL	3.809081414	1.493999925	-1.350263049	1.29E-167	3.80E-166
CA9	2.105428707	0.83993815	-1.325759021	2.97E-69	5.20E-69
PROM2	5.102500449	0.539001385	-3.24284352	2.75E-157	1.59E-156
NOS2	0.171350737	0.498067178	1.539387945	1.76E-37	2.50E-37
MT3	1.298373979	0.423496369	-1.616284488	2.63E-97	5.38E-97
ALB	0.946460434	0.296042738	-1.676736732	3.24E-115	8.14E-115
SRXN1	2.644855994	1.093893951	-1.273716293	8.60E-155	4.51E-154
GPX2	3.222664833	0.586921476	-2.457014747	9.65E-136	3.01E-135
CHAC1	0.72617889	2.723315712	1.906967346	2.55E-120	6.68E-120
SLC7A5	3.406532295	7.556078028	1.149333716	4.16E-158	2.53E-157
XBP1	5.389831879	0	1.358426985	5.92E-186	1.37E-183
ATF3	5.108191447	2.501198634	-1.030192961	9.41E-127	2.62E-126
ZFP69B	0.24111542	1.40524403	2.543024863	1.63E-161	1.26E-160
GDF15	1.360054056	6.65575073	2.29093741	3.15E-143	1.10E-142
TUBE1	3.065373465	1.461744026	-1.068372158	8.04E-164	1.03E-162
RGS4	0.172758944	1.102595678	2.67407154	5.57E-124	1.51E-123
BLOC1S5-TXNDC5	2.270127528	0.166691668	-3.767519446	4.46E-165	6.86E-164
SLC2A6	0.661548558	2.391072852	1.853739127	1.69E-141	5.65E-141
SLC2A12	1.054663445	0.301338811	-1.807324291	1.08E-118	2.80E-118
SLC2A14	0.215078088	0.085373971	-1.332992354	2.53E-52	3.98E-52
ALOX12	3.074077773	1.068310759	-1.524822295	7.01E-142	2.42E-141
ACSF2	5.272515903	2.557740659	-1.043621551	8.17E-162	7.26E-161
HBA1	5.869977348	0.133543156	-5.457976992	8.04E-169	9.28E-167
PLIN4	2.43673262	0.560742829	-2.119536784	4.05E-117	1.04E-116
RRM2	1.140175128	4.03701268	1.824032683	7.33E-162	6.77E-161
AURKA	0.978068557	3.929622953	2.006383395	3.80E-167	8.78E-166
CYBB	0.693968451	3.578579102	2.366444887	7.40E-153	3.42E-152
DUOX1	5.260645029	0.843540702	-2.640710117	2.30E-161	1.71E-160
DUOX2	1.93452121	0.12617319	-3.938499249	2.40E-163	2.75E-162
GLS2	1.616236288	0.080402128	-4.329260629	3.68E-168	2.13E-166
ALOX12B	5.144547757	0.422419889	-3.60629459	7.14E-156	4.02E-155
ALOX15B	3.262166716	0.97144888	-1.747620529	2.29E-123	6.14E-123
ALOXE3	5.251275389	0.293465655	-4.161404279	2.50E-163	2.75E-162
EGFR	4.994341403	0.976902144	-2.354008484	6.72E-164	9.13E-163

CDKN2A	0.578668699	2.445476736	2.079306227	3.09E-52	4.82E-52
MYB	1.50068396	0.675067831	-1.152515805	1.10E-107	2.46E-107
TGFBR1	1.886247392	4.34464929	1.203720818	3.76E-167	8.78E-166
IFNG	0.040285597	0.908429361	4.495038299	2.65E-95	5.33E-95
MIOX	0.029606503	0.149330253	2.334520464	1.84E-42	2.69E-42
FBXW7	5.114633241	1.973353502	-1.373981371	1.32E-167	3.80E-166
